# Prevalence and correlates of disordered eating in a general population sample: the South East London Community Health (SELCoH) study

**DOI:** 10.1007/s00127-014-0822-3

**Published:** 2014-01-20

**Authors:** F. Solmi, S. L. Hatch, M. Hotopf, J. Treasure, N. Micali

**Affiliations:** 1 Behavioural and Brain Sciences Unit, Institute of Child Health, University College London, 30 Guilford Street, WC1N 1EH, London, UK; 2 Psychological Medicine, Institute of Psychiatry, King’s College London, London, UK; 3 Eating Disorders Department, Institute of Psychiatry, King’s College London, London, UK

**Keywords:** Disordered eating, Ethnic minorities, Comorbidity, Mental illness

## Abstract

**Purpose:**

Disordered eating has been shown to be more prevalent than full eating disorders diagnoses. However, research on its prevalence, socio-demographic, psychological correlates, and patterns of service use in multi-ethnic samples is still limited. This paper explores these associations in a South London-based (UK) sample.

**Methods:**

The South East London Community Health (SELCoH) study is a general population survey (*N* = 1,698) of individuals aged 16+. Disordered eating was defined as ≥2 positive answers at the SCOFF questionnaire. Crude and adjusted logistic and multinomial logistic regression models were fit to investigate associations between socio-demographic characteristics, disordered eating, psychiatric comorbidity, and service use.

**Results:**

A total of 164 (10 %) participants reported disordered eating and the majority were from ethnic minorities. In adjusted models, Asian ethnicity was associated with purging, loss of control eating and preoccupation with food. Individuals with disordered eating had higher odds of screening positive for post-traumatic stress disorder and personality disorders and of having anxiety/mood disorders, suicidal ideation/attempts, hazardous levels of drinking, and used drugs in the previous year. Only 36 % of individuals with disordered eating had sought professional help in the previous 12 months mostly through their general practitioner (27.4 %), followed by psychotherapists (12.8 %) and mental health specialists (5.5 %).

**Conclusion:**

This study found a high prevalence of disordered eating, especially amongst ethnic minorities, and associations with a number of psychiatric conditions. Overall few participants accessed specialist services. These findings suggest that both disordered eating manifestations amongst ethnic minorities and access to care need better investigation.

**Electronic supplementary material:**

The online version of this article (doi:10.1007/s00127-014-0822-3) contains supplementary material, which is available to authorized users.

## Introduction

### Eating disorders epidemiology

Eating disorders (ED) are a set of psychiatric conditions traditionally considered of low prevalence in the general population [1–5]. However, increasing evidence suggests that sub-threshold presentations of ED are more prevalent in the population than previously assumed. Whilst lifetime prevalence of anorexia nervosa (AN), bulimia nervosa (BN), and binge eating disorder (BED) has been estimated to range between 0.3 and 0.9 %, 0.9 and 1.5 %, and 1.9 % and 3.5 % [1], respectively, the lack of uniform sets of diagnostic criteria for their definition has caused prevalence figures for eating disorders not otherwise specified (EDNOS) to be generally scant. Nevertheless, a growing body of evidence suggests that EDNOS are more prevalent than AN and BN [6–8] and that their incidence has increased [9]. Prevalence of EDNOS has been estimated in the range 2–5 % [1, 7, 10, 11] and they seem to account for the majority of all ED cases diagnosed in outpatients and general population settings, with proportions ranging from 50 to 90 % [6, 8, 11–13]. A recent UK study employing General Practice Research Database (GPRD) data from 2000 to 2009 estimated that the incidence rate of EDNOS in women aged 10–49 has increased from 17.7 per 100,000 in 2000 to 28.4 per 100,000 in 2009 and in men from 3.4 per 100,000 in 2000 to 4.2 per 100,000 in 2009 [9]. A study of 2,520 participants found that 3.9 % (5.9 % women and 1.5 % men) of participants reported any ED behaviours, with higher prevalence among younger and overweight to obese participants [14]. Another study using data from the National Adult Psychiatric Comorbidity Survey 2007 (*N* = 7,001) found 9.1 % of women and 3.4 % of men reporting disordered eating [15].

### Comorbidity

Substantial associations with Axis I (i.e. mood and anxiety disorders, substance abuse) [16] and Axis II (personality disorders) psychiatric comorbidity have been documented in all ED [17]. There is a good evidence that AN, BN, and BED are co-morbid with emotional disorders, both depression [18–24] and anxiety [18], substance use disorders [16, 20–22], and suicidality [16, 20–22]. According to several studies, individuals with EDNOS experience similar co-morbid conditions of those with full-fledged diagnoses [4, 8, 12, 15, 21, 24–26] having increased odds of reporting mood [8, 24, 27–30] and anxiety disorders [8, 28–30], substance use [8, 21, 24, 30, 31], and suicidality [8, 27]. Individuals with disordered eating are more likely to have higher levels of anxiety and depression [15, 25, 32] compared to healthy individuals. More heterogeneous evidence exists with respect to substance abuse; some studies report evidence of an association with disordered eating [25], and others only find evidence for weaker associations in general population samples than those typically seen in clinical ones [33].

### ED in minority groups and help-seeking behaviours

The majority of research in ED to date has employed samples consisting mainly of women of White European or North American ethnicity. Little is still known about the manifestation and epidemiology of disordered eating among men [34], and, despite a surge in research in the past decade, contradictory results exist when looking at disordered eating in ethnic minorities.

Existing research suggests that ED cognitions and behaviours might have different presentations in males than females [34]. Several studies have found that individuals from Black and other minority groups show fewer body image issues [35, 36] and lower prevalence of AN [2], whilst contrasting results have been found with regards to binge eating, and compensatory behaviours [35]. Nevertheless, most studies have focused on comparisons between Black and White populations, with comparatively less attention paid to Hispanic and Asian groups. A recent meta-analysis uncovered differences in body dissatisfaction between women of White and Black ethnicity, but not between women of White, Asian and Hispanic ethnicity [37]. Hence, more research is needed in multi-ethnic samples to further disentangle these trends.

It is extensively reported in literature that ED patients are reluctant to disclose their symptoms and seek help both at the general medical level and the specialised one. Hudson and colleagues found that only 50–63.2 % of ED participants had sought help for any emotional problems in their lifetime mostly through general medical services, whereas only about 43 % had sought help for ED specifically. Fewer people (15.6 % with BN and 28.5 % with BED) sought help through general medical services in the 12 months prior to assessment [4].

Several studies report that members of ethnic minorities suffering from an ED are less likely to present to services to seek help and when they do, they are less likely to receive treatment [38–40]. An American study found that participants Hispanic and Native American ethnicity were less likely than those of White ethnicity to be referred for specialist care evaluation [38]. Similarly, a South London-based one found ethnic minority patients to be under-represented in the clinic and less likely to be offered treatment despite being more likely to be diagnosed with an ED [40].

Differences in ED manifestations across different populations (e.g. men, ethnic minorities) coupled with evidence that sub-threshold conditions are often co-morbid with other psychiatric disorders highlight the need for research on disordered eating (i.e. ED behaviours and cognitions) beyond mere diagnoses. This, in turn, could help health professionals identifying ‘at risk’ individuals, who might otherwise go undetected.

The aims of this paper were: to estimate prevalence and correlates of disordered eating in a general population sample based in South East London, UK; to explore differences across gender, ethnicity and body mass index (BMI) groups; and to explore patterns of service use among individuals with disordered eating. Based on findings of previously mentioned studies, we hypothesised: that disordered eating would be highly prevalent [15]; that disordered eating would be similarly prevalent amongst ethnic minority and White ethnic groups; and that most participants would have sought help for a mental health problem through their GP, although fewer from specialist services.

## Methods

### Study population

The SELCoH study population was recruited in the two London boroughs of Lambeth and Southwark. The small user postcode address file (PAF) was used as sampling frame to identify households. Households receiving more than 50 items of mail per day were excluded to avoid including business addresses. All eligible individuals aged 16 years and over living within selected and participating households were invited to undertake the survey. More details on recruitment, sampling [41] and representativeness [42] of the SELCoH study population are provided elsewhere. Trained research assistants employing a purposely designed computer-based questionnaire interviewed all SELCoHI participants. Feasibility and reliability of the questionnaire were assessed during an initial pilot study [41]. All participants were interviewed between June 2008 and December 2010 and gave their consent prior to participation to the study.

### Measures

#### Exposure

Disordered eating was assessed using the SCOFF questionnaire, a 5-item questionnaire developed by Morgan and colleagues [43, 44] as a screening tool for ED. The acronym derives from the initials of the five main words/concepts of each of its questions:

- Do you make yourself *Sick* because you feel uncomfortably full?
- Do you worry you have lost *Control* over how much you eat?
- Have you recently lost more than *One* stone (6.35 Kg) in a 3 month period?
- Do you believe yourself to be *Fat* even when other say you are too thin?
- Would you say that *Food* dominates your life?

The SCOFF has been validated in a number of studies and settings, showing good sensitivity and specificity, although low positive predictive value (PPV), especially in general population settings [43, 45–48]. When investigating associations between socio-demographic characteristics (e.g. BMI, ethnicity, age) and disordered eating, both individual questions of the SCOFF and a positive screening status to the questionnaire (≥2 positive answers, as suggested by previous literature [43, 44]), were used as outcome measures.

#### Psychological correlates

##### Psychiatric morbidity

The revised Clinical Interview Schedule (CIS–R) [49], a 14-item structured questionnaire designed to be used by lay interviewers’ in non-clinical settings to assess depressive and anxiety symptoms [50], was used to assess psychiatric comorbidity. Its questions, scored on a 0–4, or 0–5 scale (in the case of the depressive ideas scale), cover a number of neurotic symptoms. Scores above a 12-point cut-off indicate suspected presence of a common mental disorder (CMD). An algorithm can be employed to derive primary and secondary diagnoses [49, 51]. To increase power of analyses, we created a three-level variable from the CIS-R-derived primary diagnoses indicating ‘no diagnosis’, ‘non-specified neurotic disorders’ (i.e. all those participants who scored above the 12 point CIS-R cut-off, but did not reach a full diagnosis) and ‘mood, anxiety and mixed mood and anxiety disorders’ grouped together.

##### Personality disorder

The standardised assessment of personality-abbreviated scale (SAPAS) [52] was employed to screen for symptoms of personality disorders (PD). The SAPAS is an 8-item questionnaire in which each question addresses a personality aspect and is scored 0–1. Previous studies have identified a cut-off of 3 or 4 as maximising sensitivity and specificity of the instrument [52, 53], although the extent of PPV of the SAPAS in a general population sample has not been validated yet. In this study, a cut-off of 4 was used to define individuals with a PD.

##### Post-traumatic stress disorder

The primary care post-traumatic stress disorder scale (PC-PTSD), a 4-item screening measure designed for use in primary care and other medical settings, was employed to screen for PTSD [54].

##### Suicidal ideation and/or attempt

A variable was derived indicating whether a participant reported having had suicidal ideation or acts. Suicidal ideation was assessed with the question: “have you ever thought of taking your own life, even if you would not really do it?”, and suicide attempts with the question: “have you ever made an attempt to take your life, by taking an overdose or in some other way?” Both items had been previously employed by the 2000 and 2007 British National Psychiatric Morbidity surveys [55, 56]. The rationale for collapsing the two questions was provided by the relatively low prevalence of suicidal attempts [42], the hypothesised low prevalence of disordered eating in the sample and, thus, by the need to increase the power of our analyses. Moreover, it is possible to conceptualise suicidal ideation and attempts as elements of a continuum of severity of suicidal behaviours.

##### Alcohol use

Alcohol consumption was investigated with the alcohol use disorders identification test (AUDIT) [57], a screening tool created by the World Health Organization (WHO) to assess excessive drinking. The AUDIT comprises 10 questions scored on a 0–4 point Likert scale (0 = never, 1 = less than monthly, 2 = monthly, 3 = weekly, 4 = daily or almost daily). Suggested cut-offs are: 0–7 ‘healthy drinking’, 8–15 ‘hazardous drinking’, 16–19 ‘hazardous and harmful drinking’, and ≥20 ‘alcohol dependence’ [1]. In this study, ‘hazardous and harmful drinking’ and ‘alcohol dependence’ were merged to increase power of the analyses.

##### Any drug use

Participants were asked about drug consumption in the previous 12 months. Drugs included in the questionnaire were: Cannabis, Amphetamines, Cocaine, Ecstasy, Acid or LSD, Tranquillisers, Crack, and Heroin. From the individual answers, a summary variable was generated indicating whether the participant had tried at least one drug in the previous 12 months. The rationale for collapsing cannabis use (more frequent) with that of other drugs was that use of any illicit substance could be understood as a marker of risk-taking behaviour.

##### Smoking

Participants’ smoking status was coded as a three-level categorical variable indicating ‘never smoked’, ‘ex-smoker’, and ‘current smoker’.

#### Help-seeking

All participants were asked whether they had sought help from a GP or a therapist for a problem with anxiety and depression in the previous year. Those answering positively to this first screening question were asked follow-up questions about whether they had seen a GP, therapist or counsellor, or a mental health specialist. Participants were also asked whether they had received a series of diagnoses. Each diagnosis was asked about individually to account for the possibility of multiple diagnoses.

#### Socio-demographic characteristics

Information on participants’ anthropometric measures was collected at the time of the interview. BMI was calculated from objective measurements of height and weight and recoded as a 4-level categorical variable indicating whether the participant was underweight (BMI < 18.5), normal weight (18.5 ≤ BMI < 25), overweight (25 ≤ BMI < 30), or obese (BMI ≥ 30). Age, measured continuously, was subsequently coded into a categorical variable indicating six age groups (16–24; 25–34; 35–44; 45–54; 55–64; 65+) for ease of interpretation and in line with previous papers [41].

Information was also collected on ethnicity, education level, and marital status. To increase statistical power of analyses, ethnicity was summarised in four categories according to whether the participant was from a White, Black, Asian or any other ethnic background. White ethnic background referred to participants of White British background; Black ethnic background those of both Black African (*N* = 234, 13.8 %) and Black Caribbean (*N* = 143, 8.4 %) ethnicity; and Asian ethnic background those of Indian, Pakistani, Bangladeshi, or Chinese ethnicity. Participants were assigned to the ‘any other ethnic background’ if they did not identify themselves with any of the categories described above. Education was coded as a 3-level categorical variable indicating whether the participant had no qualifications, completed GCSE (national exams undertaken at age 16) and/or A-levels (national exams undertaken at age 18), or had a higher degree or above. Finally, participants’ marital status was recoded as single, married or cohabiting, divorced or separated, or widowed.

#### Data analyses

Analyses were conducted accounting for household clustering and weighting for non-response within household. In survey studies, weighting data for non-response are necessary to correct for non-response bias, and generate accurate prevalence estimates and robust standard errors. How inverse probability weights were calculated for the SELCoHI sample is described elsewhere [41].

All analyses were run on individuals with complete outcome and exposure data.

Associations between socio-demographic variables and any disordered eating were described using unweighted frequencies, although weighed prevalence and 95 % CIs were calculated and tested for differences with Pearson’s *χ* ^2^ tests with Rao–Scott correction for categorical data from survey samples [58].

Missing data on socio-demographic variables was assumed to be missing at random (MAR). Missing values for categorical variables were imputed from multinomial logistic models. Distribution of imputed values was visually inspected to ensure comparability with the observed value.

Univariate and multivariate logistic and multinomial logistic regressions were employed to calculate odds ratios (OR) and 95 % confidence intervals (CIs) for the association between disordered eating and behavioural and psychological outcomes, and help-seeking behaviours. In multivariate models, analyses were adjusted for socio-demographic covariates thought a priori to be potential confounders of the association between the exposure and outcomes of interest. Help-seeking outcomes were also adjusted for mood and anxiety disorders, as the question asked if the participants had sought professional help for ‘problems with anxiety and depression in the previous year’.

All analyses were performed using Stata12.

## Results

### Sample characteristics

A total of 1,698 individuals (age 16–90) took part in SELCoHI. The majority of participants included in the analyses were women (56.6 %), were educated at least to GCSE/A-levels (45.4 %) and were from a white background (62.4 %) (Table 1). However, the sample reflected the ethnic diversity of the studied area and included 22 % of participants from a Black ethnic background (African or Caribbean), 3.7 % from Asian or British Asian background, and 12 % from mixed or other backgrounds. Mean age for the sample was 39.6(SD 16.8) with the age group 25–34 years being the most represented (24.1 %).

**Table 1:** Socio-demographic characteristics of the SELCoHI sample

	*N* (%)*
Total sample	1,645
Gender	
Male	714 (43.4)
Female	931 (56.6)
Marital status	
Single	665 (40.3)
Married/cohabiting	754 (45.8)
Divorced/separated/widowed	226 (13.8)
Ethnicity (*N* = 1,643)	
White	1,024 (62.4)
Black	362 (22)
Asian	60 (3.7)
Other	197 (12)
Education (*N* = 1,626)	
No qualification	208 (12.8)
GCSE/A-level	738 (45.4)
Degree level or above	680 (41.8)
BMI (*N* = 1,586)	
Underweight	39 (2.5)
Normal weight	708 (44.7)
Overweight	507 (32)
Obese	332 (20.9)
Age	
16–24	352 (21.4)
25–34	396 (24.1)
35–44	328 (19.9)
45–54	252 (15.3)
55–64	152 (9.2)
65+	165 (10.4)

* Unweighted frequencies, due to missing data might not add up to the full sample

Complete data on all of the outcomes were available for 1,644 (96.8 %) participants, who were therefore included in the analyses.

Lower education level (*p* < 0.0001) and older (55+) age (*p* < 0.0001) were associated with missingness. Amongst participants included in the analyses, 95 % had complete data on all covariates; 3 % missed data on BMI, 1 % on education, less than 1 % on ethnicity, and less than 1 % on education and BMI.

### Prevalence of disordered eating

One hundred and sixty-four participants (164, 10 %) reported disordered eating. The SCOFF questions, which were more frequently endorsed, were question 2 related to perceived loss of control (*N* = 215; 13.1 %) and question 3 related to weight loss (*N* = 206, 11.7 %). (Table S1).

### Socio-demographic correlates

As shown in Table 2, more women than men (12.2 % vs. 5.9 %; *p* < 0.0001) and more participants from the youngest age group (16.1 %; *p* = 0.002) reported disordered eating. Disordered eating was most commonly reported by participants of Asian and of mixed or other ethnicity (14.8 and 16.1 %) followed by those of Black Caribbean and African (12.8 %) and White (7.9 %) ethnicity (*p* = 0.001). Finally, more participants from the obese BMI group (13.9 %; *p* = 0.05) reported disordered eating.

**Table 2:** Prevalence estimates for disordered eating across socio-demographic characteristics

Socio-demographic characteristics	N***	Disordered eating		*p* value**
		*n*	Prevalence* (95 % CI)	
Total	1,645	164	10.1 (8.6–11.8)	
Gender				
Male	714	42	5.9 (4.3–7.9)	<0.0001
Female	931	122	12.2 (10.2–14.5)	
Marital status				
Single	665	74	11.7 (9.3–14.7)	0.13
Married/cohabiting	754	63	8.3 (6.5–10.7)	
Divorced/separated/widowed	226	27	11.2 (7.7–16.1)	
Ethnicity***	1,643			
White	1,024	80	7.9 (6.2–9.8)	0.001
Black	362	46	12.8 (9.6–17)	
Asian	60	9	14.8 (7.5–27.2)	
Other	197	29	16.1 (11.3–22.4)	
Education***	1,626			
No qualification	208	25	10.5 (7–15.4)	0.02
GCSE/A-level	738	87	12.4 (10–15.2)	
Degree level or above	680	50	7.4 (5.6–9.9)	
Body mass index (BMI)***	1,586			
Underweight	39	4	11.4 (4.4–26.3)	0.05
Normal weight	708	58	8.2 (6.3–10.6)	
Overweight	507	48	9.6 (7.2–12.7)	
Obese	332	47	13.9 (10.4–18.3)	
Age				
16–24	352	52	16.1 (12.2–20.9)	0.002
25–34	396	42	11.7 (8.6–15.6)	
35–44	328	28	9.4 (6.6–13.2)	
45–54	252	24	9.9 (6.7–14.5)	
55–64	152	9	5.6 (2.9–10.7)	
65+	165	9	5.5 (2.8–10.4)	

* Weighted percentages to account for survey design

** Pearson’s *c* test with Rao & Scott correction for survey data

*** Unweighted frequencies, due to missing data might not add up to the full sample

The distribution of socio-demographic variables across individual SCOFF questions showed that mostly participants of Asian and other ethnic backgrounds endorsed the question on purging behaviours; more men than women and more participants of Black ethnicity endorsed the weight loss question. Participants of Asian ethnicity also mostly endorsed the loss of control eating and preoccupation with food questions. (Table S2).

BMI was associated with loss of control and preoccupation with food. As shown in Table S2, a greater proportion of participants endorsing the loss of control question were from the overweight and obese categories, whereas no differences were shown for preoccupation with food. More underweight participants endorsed the question on purging behaviours.

Strong associations were found between Black, Asian and other ethnicities and disordered eating compared to white ethnicity in the univariate model. However, once adjusting for other socio-demographic characteristics only ‘other’ ethnic background remained strongly associated with disordered eating (Table 3). When looking at associations between ethnic groups and individual SCOFF answers (Table S3), participants of Asian ethnicity were more likely to report purging (OR 3.7, 95 % CI 1.4–9.8), loss of control (OR 2.2, 95 % CI 1.1–4.2) and preoccupation with food (OR 3.1, 95 % CI 1.5–6.3); and participants of other ethnic backgrounds were more likely to report purging (OR 2.7, 95 % CI 1.4–5.4), body image distortion (OR 2.2, 95 % CI 1.2–3.9), and preoccupation with food (OR 2.4, 95 %CI 1.5–3.9) compared to participants of White ethnicity. No associations were found between Black ethnicity and any of the SCOFF questions.

**Table 3:** Logistic regression analyses of the association between ethnicity, BMI and disordered eating (SCOFF screen positive) (odds ratios, 95 % confidence intervals, *p* value)

	*N*	*n* ^1^	Crude OR (95 % CI)	*p* value	Adjusted OR^2^ (95 % CI)	*p* value
Total	1,645	164				
Ethnicity						
White	1,024	80	1.0	–	1.0	–
Black	362	46	1.7 (1.2–2.6)	0.008	1.2 (0.8–1.9)	0.4
Asian	60	9	2.0 (0.9–4.6)	0.08	1.9 (0.9–4.6)	0.1
Other	197	29	2.3 (1.4–3.6)	0.001	1.8 (1.1–3.0)	0.02
BMI						
Underweight	39	4	1.3 (0.4–3.8)	0.6	1.6 (0.5–4.8)	0.3
Normal weight	708	58	1.0	–	1.0	–
Overweight	507	48	1.2 (0.8–1.7)	0.4	1.5 (0.9–2.2)	0.07
Obese	332	47	1.7 (1.1–2.6)	0.01	2.1 (1.3–3.4)	0.002

Multiple imputation models

^1^Number of SCOFF positive individuals

^2^Adjusted for age, gender, BMI, marital status, ethnicity, and education

In univariate models, being obese and in the multivariate models being overweight and obese were associated with disordered eating (Table 3). In multivariate models being overweight was associated with loss of control (OR 1.9, 95 % CI 1.2–2.8), whilst being obese was associated with loss of control (OR 3.2, 95 % CI 2.1–4.9), weight loss (OR 1.6, 95 % CI 1–2.5), and preoccupation with food (OR 1.9, 95 % CI 1.2–3.2) (Table S3).

Finally, as expected, female gender was associated with increased odds of disordered eating. However, females were less likely to endorse weight loss in the previous 3 months (OR 0.6, 95 % CI 0.4–0.8).

### Disordered eating and behavioural and psychological outcomes

As shown in Table 4, disordered eating was associated with having greater psychiatric comorbidity, using drugs and drinking hazardous and harmful levels of alcohol in univariate models, but not with smoking. In adjusted analyses, individuals with disordered eating had increased odds of a possible PTSD or PD diagnosis, of having anxiety, mood disorder, or both anxiety and mood disorders, sub-threshold neurotic disorders; and of suicidal ideation/attempts. Disordered eating was also associated with hazardous, and harmful and hazardous levels of drinking, as well as drug use in the previous year (Table 4).

**Table 4:** Logistic regression analyses of behavioural and psychiatric outcomes: SCOFF screen positive vs. SCOFF screen negative (odds ratios, 95 % confidence intervals, *p* value)

Correlates	*N*	*n* ^1^	Crude OR (95 % CIs)	*p* value	Adjusted OR^2^ (95 % CIs)	*p* value
Total	1,645	164				
Alcohol use disorders identification test (AUDIT)						
No hazard	1,305	124	1.0	–	1.0	–
Hazardous	265	27	1.3 (0.8–2.1)	0.3	1.7 (1.0–3.0)	0.05
Hazardous and harmful	75	13	2.4 (1.3–4.6)	0.007	2.9 (1.4–6.1)	0.003
Smoking status						
Non-smoker	502	53	1.0	–	1.0	–
Current	704	76	1.1 (0.7–1.6)	0.8	1.2 (0.7–1.8)	0.5
Ex-smoker	439	35	0.7 (0.5–1.2)	0.2	1.1 (0.6–1.8)	0.8
Primary diagnosis						
No diagnosis	1,212	76	1.0	–	1.0	–
Non-specified neurotic disorder	108	15	2.3 (1.3–4.2)	0.006	2.1 (1.1–3.9)	0.02
Anxiety/mood/anxiety + mood disorder	325	73	4.1 (2.8–5.9)	<0.0001	4.1 (2.7–5.9)	<0.0001
Any drug use last year						
No	1,286	120	1.0	–	1.0	–
Yes	359	44	1.4 (1.0–2.1)	0.05	1.6 (1.1–2.5)	0.03
Standardised assessment of personality-abbreviated scale (SAPAS)						
No	1,411	109	1.0	–	1.0	–
Yes	234	55	3.4 (2.4–4.9)	<0.0001	3.2 (2.1–4.8)	<0.0001
Post-traumatic stress disorder (PTSD)						
No	1,562	139	1.0	–	1.0	–
Yes	83	25	4.7 (2.8–7.7)	<0.0001	4.5 (2.7–7.6)	<0.0001
Suicide attempt or ideation						
No	1,290	101	1.0	–	1.0	–
Yes	355	63	2.4 (1.7–3.5)	<0.0001	2.5 (1.7–3.6)	<0.0001

Multiple imputation models

^1^Number of SCOFF positive individuals

^2^Adjusted for age, gender, BMI, marital status, ethnicity, and education

### Disordered eating and service use

As shown in Table 5, about a third (*N* = 59, 36 %) of individuals with disordered eating had sought professional help in the year prior to the interview for an anxiety or depression-related problem; the majority saw a GP (*N* = 45, 27.4 %), followed by a therapist (*N* = 21, 12.8 %) and mental health specialist (*N* = 9, 5.5 %). About a third of individuals with disordered eating reported that they did not seek help although they thought they needed it (*N* = 52, 31.7 %). Strong associations were found between disordered eating and service use; participants with disordered eating had increased odds of having sought help and of having seen a GP, a mental health therapist or specialist in adjusted models.

**Table 5:** Proportion of SCOFF positive and negative participants who sought help for their mental health concerns and odds ratios (OR) and 95 %CI of the association between disordered eating and help-seeking behaviours

Outcome indicator	SCOFF negative		SCOFF positive		*p* value	Crude OR (95 % CI)	*p* value	Adjusted OR^1^ (95 % CI)	*p* value
	*n*	Prevalence (95 % CI)	*n*	Prevalence (95 % CI)					
Sought help									
No	942	62.6 (59.9–65.3)	53	31.8 (24.5–39.9)		1.0		1.0	
No, though I should have	314	21.6 (19.4–24)	52	31.9 (25.1–39.5)	<0.0001	2.9 (1.9–4.4)	<0.0001	2.1 (1.3–3.3)	0.002
Yes	225	15.7 (13.8–17.8)	59	36.3 (29.1–44.3)		4.6 (3.1–7.1)	<0.0001	3.2 (1.9–5.2)	<0.0001
Seen a GP									
No	1,319	88.6 (86.7–90.3)	119	72.6 (64.9–79.1)	<0.0001	1.0		1.0	
Yes	162	11.4 (9.7–13.2)	45	27.4 (20.9–35.1)		2.9 (1.9–4.4)	<0.0001	2.9 (1.9–4.4)	<0.0001
Seen a therapist									
No	1,387	93.7 (92.2–94.9)	143	86.9 (80.6–91.5)	0.002	1.0		1.0	
Yes	94	6.3 (5.1–7.8)	21	13.1 (8.6–19.5)		2.2 (1.3–3.7)	0.003	2.5 (1.5–4.2)	0.001
Seen a mental health specialist									
No	1,459	98.4 (97.6–94.7)	155	94.7 (90–97.2)	0.001	1.0		1.0	
Yes	22	1.6 (1–2.4)	9	5.3 (2.8–9.9)		3.5 (1.6–7.9)	0.003	3.2 (1.4–7.1)	0.005
ED diagnosis									
N/A	1,255	84.8 (82.8–86.6)	105	64 (56.3–71.1)	<0.0001				
No	215	14.6 (12.9–16.5)	47	29.5 (22.8–37.3)					
Yes	1	–	7	4.4 (2.1–8.9)					

Multiple imputation models

^1^Adjusted for age, gender, BMI, marital status, ethnicity, and education and CMD

## Discussion

This study aimed to determine the prevalence of disordered eating and its correlates in a South East London-based (UK) general population sample. Ten per cent of participants reported disordered eating in the year prior to interview. Disordered eating was more common amongst females and ethnic minorities.

Two recent studies employed the SCOFF questionnaire to estimate disordered eating in the general population [15, 25]. McBride et al. [15] employing data from the UK National Adult Psychiatric Morbidity Survey 2007 found a prevalence of 6.3 %. This figure was slightly lower than the one we reported. Differences in the population studied, a UK-wide sample vs. an inner-city sample in our case, are likely to account for this finding, as rates of ED and psychiatric disorders are known to be higher in urban settings [59, 60]. A higher prevalence of disordered eating (29.4 % in girls and 14.4 % in boys) was reported by Herpertz-Dahlmann et al. [25] in a sample of 1,895 German adolescents. The difference in prevalence between the latter study and ours could be due to the different age groups of the samples. Some ED are more typical of adolescent age, therefore it is possible that a higher prevalence of disordered eating is detected in younger populations.

In this sample, the most commonly endorsed disordered eating symptoms were loss of control eating, weight loss and preoccupation with food-related thoughts. Although disordered eating was more common amongst women, there were no differences in the prevalence of males or females endorsing each of the SCOFF questions apart from the weight loss question, which was associated with male gender. Recent literature has suggested an increasing awareness among men of body image issues due to societal pressures to adhere to specific models, which might reflect in higher rates of ED behaviours amongst men than those of previous decades [34]. However, the questionnaire was not able to detect further differences between genders, possibly due to the broad nature of the questions asked.

Previous literature had also argued that ethnic minorities might have a decreased risk of developing ED due to lower societal and cultural preferences for thin figures [35, 36]. Older studies reported mixed findings; results have showed lower levels of bulimic symptoms among Black and Asian minorities [61, 62], higher levels of purging behaviours in Black minorities only [61, 63] and higher prevalence of binge-eating in ethnic minorities’ women [64]. Our results are in line with some recent studies, showing Asian ethnicity to be associated with ED behaviours. A qualitative study investigating ethnic differences in women’s responses of mainstream beauty standards, found that Asian women were more likely than Black women to endorse them in a similar fashion to white women, as well as experiencing greater body dissatisfaction [65]. Moreover, a systematic review focusing on children’s mental health issues in the UK has highlighted that children and adolescents from an Asian background had the highest levels of ED behaviours amongst all other ethnic minorities in six out of the seven studies included [66]. Finally, a recent South London-based study [40] on a clinical sample of 648 patients assessed for an ED found that a greater proportion of individuals diagnosed with BN were from ethnic minority groups, of which Asians were the most prevalent. Our findings thus suggest that disordered eating and ED manifestation in ethnic minorities, usually underplayed, need further investigation in multi-ethnic and inner-city settings, where they are more prevalent. It is possible to speculate that environmental and societal factors might play a distinct role in the aetiology of disordered eating in these groups. Stressful life events and discrimination might also influence this association, although this was not investigated in this study. Further research should focus on better understanding these links.

Finally, we found disordered eating associated with the ‘other ethnic background’ category; however, given the broad range of ethnicities included it is not possible to make specific inferences about associations with some ethnic backgrounds over others.

Individuals of overweight and obese BMI categories (52.9 % of the sample) had increased odds of reporting disordered eating, and loss of control and preoccupation with food, in particular. Previous studies have shown that binge eating is associated with higher BMI [64] and that loss of control eating is associated with anxiety and overweight BMI status [67]. It is therefore possible that disordered eating patterns precede and are associated with higher BMI in these individuals or that weight loss reflects attempts at dieting. However, it is possible that, in some individuals, these episodes of disordered eating behaviours might signal clinically significant conditions. In fact, disordered eating has been shown to be more prevalent in obese groups [68] and that the latter often show high levels of eating restraint [69]. Recent studies have also suggested that increased rates of obesity in society and subsequent attempts to lose weight might act as risk factor for the development of disordered eating, unhealthy weight control practices (i.e. self-induced vomiting, laxatives, fasting) and, possibly, eating disorders [70, 71]. The strong associations found between disordered eating and several psychopathology measures suggest that this hypothesis could be plausible. From this study it is, however, not possible to disentangle these associations and risk mechanisms, although it is interesting to have replicated previous findings suggesting an association between BMI, and disordered eating and loss of control. Future studies should address these hypotheses using longitudinal designs.

We also found an association between disordered eating and comorbidity with possible PD and PTSD; suicidal ideation and attempts; anxiety, mood, or mixed mood and anxiety disorders; and hazardous levels of drinking and drug use. Our results confirm previous findings that ED behaviours, irrespectively of whether they reach full diagnosis, are associated with important comorbidity. This finding is in line with those of previous literature showing that individuals with sub-threshold diagnoses have comparable levels of mood and anxiety disorders [4, 21, 24] and suicidality [8, 25, 27, 72] to those of individuals with full-threshold ED diagnoses when compared against healthy ones. Individuals exhibiting purging behaviours only have been shown to report these behaviours often at clinical levels [28, 30, 31]. Whilst fewer studies have investigated PD amongst disordered eating participants, evidence seems to suggest an association between the two conditions [28, 73]. Substance use has also been documented in participants with disordered eating and, in particular, amongst those with binge/purge type behaviours [4, 15, 21, 30, 31, 74].

Finally, this study found that although about 30 % of participants with disordered eating reported having seen a GP for problems such as anxiety or depression, only 13 % had seen a mental health therapist and 5 % a mental health specialist. These findings might reflect the UK ‘gatekeeper’ system where mental health specialists need GP referral. However, it could also reflect the inability of GPs to identify patients with an ED, especially those with binge-eating disorder, who might be otherwise be referred to dieticians, for instance. Finally, it could also imply that the less severe cases might be less likely to attend services or that members of ethnic minorities are less likely to receive an ED diagnosis, as it has been documented in previous literature [38–40]. Since only 7 (4.4 %) individuals with disordered eating reported having been diagnosed with an ED, the last two hypotheses seem plausible. Nevertheless, disordered eating was associated with service use.

These results should be interpreted in light of some limitations. ED are highly co-morbid with a number of psychiatric conditions, especially with anxiety and mood disorders. This study, given its cross-sectional nature, was unable to determine temporality, but only to establish the levels of comorbidity associated with disordered eating.

Although 37.6 % of our sample was from a non-White ethnic background it was not possible to investigate in detail differences amongst ethnic minorities, due to low numbers and a high proportion (12 %) of participants from ‘other’ ethnic background, which was not possible to further identify. Future studies should aim at recruiting bigger samples of participants from ethnic minorities to increase power of analyses and achieve higher precision of results.

Finally, some of the measures employed to explore comorbidity were screening measures, therefore it was not possible to explore more in detail, the associations highlighted (e.g. which PD was associated with disordered eating). Moreover, the SCOFF questionnaire, a screener measure for eating disorders, was used to define disordered eating. From the SCOFF it is neither possible to derive ED diagnoses nor to estimate reliable sub-scales related to specific ED behaviours, as it is the case for other questionnaires (i.e. EDE-Q). However, the SCOFF has been validated in the general population with good values of sensitivity and specificity [43, 44, 46, 75] and other studies have recently employed it to assess prevalence of disordered eating in large general population samples [15, 25]. Moreover, it captured a group of participants who showed strong associations with co-morbid behaviours which is consistent with evidence showing that that sub-threshold ED presentations might have a similar psychiatric burden to that of full diagnoses.

This study had important strengths. Firstly, it employed a large general population sample representative of the population in the study catchment area, which includes a high proportion of ethnic minorities, ensuring generalizability of the results.

Secondly, these results have significant clinical implications as they suggest the importance of screening for disordered eating in the community, not only to potentially detect individuals with eating disorders, but also, given the comorbidity found with other psychiatric disorders, to identify participants at risk of having or developing further conditions. Findings on lower rates of specialist care attendance amongst disordered eating participants point to the need for better referral systems at GP level, whereas high proportions of participants who reported not having sought help despite feeling the need suggest perhaps the necessity for better community education on mental health to reduce the stigma associated with it. Future studies should employ longitudinal interventions to assess the impact of screening programmes at community (e.g. schools, GP) level on referral rates and mental health morbidity amongst eating disordered participants.

## Electronic supplementary material

Below is the link to the electronic supplementary material.

Supplementary material 1 (DOCX 88 kb)
