# Supplementary material for: Prevalence and correlates of disordered eating in a general population sample: the South East London Community Health (SELCoH) study
Source: Soc Psychiatry Psychiatr Epidemiol. 2014 Jan 20;49(8):1335–46. doi: 10.1007/s00127-014-0822-3 (PMC4108843; doi:10.1007/s00127-014-0822-3)
Supplement: Supplementary file 1 — Supplementary material 1 (DOCX 88 kb) [file 127_2014_822_MOESM1_ESM.docx]

**Title: Prevalence and correlates of disordered eating in a general population sample: the South East London Community Health (SELCoH) study.**

*Authors: Solmi, F.^1^; Hatch, S.L.^2^; Hotopf, M.^2^; Treasure, J.^3^, Micali, N.^1^*

Journal: Social Psychiatry and Psychiatric Epidemiology

*^1^Behavioural and Brain Science Unit, Institute of Child Health, University College London, London, UK (Ms Francesca Solmi; Dr Nadia Micali)*

*^2^ King’s College London, Psychological Medicine, Institute of Psychiatry, London, UK (Dr Stephani Hatch; Prof Matthew Hotopf)*

*^3^* *Eating Disorders Department, King’s College London, Institute of Psychiatry, London, UK (Prof Janet Treasure)*

**Corresponding author:**

Francesca Solmi, MSc

Behavioural and Brain Science Unit,

Institute of Child Health, University College London

30 Guilford Street, London

WC1N 1EH

Tel: +44(0)2079052166

Fax: +44(0)2078317050

Email: [francesca.solmi.10@ucl.ac.uk](mailto:francesca.solmi.10@ucl.ac.uk)

**Supplementary Material**

Table S1: Prevalence and 95%CIs of 'yes' answers to each SCOFF item

| **SCOFF items** | **N** | **Prevalence (95%CI)*** |
| --- | --- | --- |
| Made yourself **S**ick | 53 | 3.1(2.4-4.1) |
| Loss of **C**ontrol | 215 | 13.1(11.8-13.3) |
| Lost **O**ne Stone | 206 | 11.7(10.3-13.4) |
| Believed to be **F**at | 113 | 6.9(5.7-8.3) |
| **F**ood Dominates Life | 154 | 9.6(8.2-11.3) |

*Weighted percentages to account for survey design

Table S2: Prevalence and 95% CIs of positive answers to each SCOFF item across socio-demographic characteristics of SELCoH sample (N=1,645).

| **Socio-demographic**  **characteristic** | **Scoff Sick**  **(Item 1)** | | | **Scoff Loss of Control**  **(Item 2)** | | | **Scoff weight loss**  **(Item 3)** | | | **Scoff body image distortion**  **(Item 4)** | |  | **Scoff food thoughts**  **(Item 5)** | | |
| --- | --- | --- | --- | --- | --- | --- | --- | --- | --- | --- | --- | --- | --- | --- | --- |
|  | n | Prevalence*  (95% CI) | p | n | Prevalence*  (95% CI) | p | n | Prevalence*  (95% CI) | p | n | Prevalence*  (95% CI) | p | n | Prevalence*  (95% CI) | p |
| **Gender** |  |  |  |  |  |  |  |  |  |  |  |  |  |  |  |
| Male (N=714) | 20 | 2.9(1.8-4.5) | 0.7 | 60 | 8.3(6.5-10.6) | <0.0001 | 105 | 14.5(12-17.4) | 0.007 | 29 | 3.7(2.6-5.3) | 0.0001 | 55 | 7.7(5.9-9.9) | 0.06 |
| Female (N=930) | 33 | 3.2(2.3-4.6) |  | 155 | 15.9(13.7-18.5) |  | 101 | 10.2(8.4-12.3) |  | 84 | 8.4(6.8-10.4) |  | 99 | 10.5(8.7-12.7) |  |
|  |  |  |  |  |  |  |  |  |  |  |  |  |  |  |  |
| **Ethnicity** |  |  |  |  |  |  |  |  |  |  |  |  |  |  |  |
| *White* (N=1,024) | 26 | 2.5(1.7-3.8) | 0.006 | 115 | 11.5(9.6-13.7) | 0.02 | 119 | 10.7(8.9-12.7) | 0.01 | 56 | 5.5(4.2-7.2) | 0.003 | 75 | 7.9(6.2-9.8) | <0.0001 |
| *Black*  (N=361) | 9 | 2.4(1.2-5.1) |  | 57 | 15.9(12.2-20.3) |  | 64 | 16.4(12.8-20.9) |  | 28 | 7.4(5.2-10.4) |  | 33 | 9.1(6.5-12.7) |  |
| *Asian* (N=60) | 5 | 7.6(3.2-16.9) |  | 11 | 22.1(13.4-34.1) |  | 4 | 6.2(2.3-15.5) |  | 5 | 8.4(3.1-21.1) |  | 12 | 20.4(11.9-32.5) |  |
| *Other* (N=197) | 13 | 6.7(3.9-11.1) |  | 32 | 16.9(12.1-23.3) |  | 19 | 9.4(5.9-14.6) |  | 24 | 13.6(8.9-19.9) |  | 34 | 17.2(12.5-23.3) |  |
|  |  |  |  |  |  |  |  |  |  |  |  |  |  |  |  |
| **Body Mass Index (BMI)** |  |  |  |  |  |  |  |  |  |  |  |  |  |  |  |
| *Underweight* (N=39) | 2 | 5.7(1.5-19.6) | 0.3 | 3 | 8.7(2.8-23.8) | <0.0001 | 2 | 4.7(1.1-17.9) | 0.13 | 3 | 6.9(2.3-19.3) | 0.4 | 5 | 12.7(5.4-27.2) | 0.02 |
| *Normal weight* (N=708) | 28 | 3.7(2.6-5.4) |  | 64 | 9.2(7.2-11.6) |  | 83 | 11.1(8.9-13.5) |  | 52 | 7.7(5.9-9) |  | 55 | 7.6(5.8-12.2) |  |
| *Overweight* (N=506) | 10 | 2.1(1.1-3.8) |  | 68 | 13.8(10.9-17.5) |  | 57 | 10.6(8.2-13.6) |  | 32 | 6.4(4.5-9) |  | 45 | 9.1(6.8-12.2) |  |
| *Obese* (N=332) | 12 | 3.8(2-6.9) |  | 72 | 21.3(17.1-26.2) |  | 56 | 14.6(11.1-19) |  | 18 | 4.9(2.9-8.1) |  | 43 | 13.7(10.2-18.1) |  |
|  |  |  |  |  |  |  |  |  |  |  |  |  |  |  |  |
| **Age** |  |  |  |  |  |  |  |  |  |  |  |  |  |  |  |
| 16-24 (N=352) | 18 | 5.5(3.5-8.6) | 0.06 | 51 | 16.1(12.2-21) | 0.01 | 66 | 18.9(15.1-23.3) | 0.0005 | 38 | 11.9(8.7-16.1) | 0.002 | 42 | 12.2(9.1-16.3) | 0.09 |
| 25-34 (N=396) | 16 | 4.2(2.6-6.7) |  | 56 | 15(11.6-19.1) |  | 45 | 11(8.3-14.5) |  | 25 | 6.7(4.6-9.8) |  | 36 | 9.7(6.9-13.4) |  |
| 35-44 (N=327) | 3 | 1.1(0.3-3.3) |  | 39 | 12.3(9.1-16.5) |  | 41 | 12.1(9-16.2) |  | 23 | 7.4(4.9-11) |  | 22 | 6.7(4.4-9.9) |  |
| 45-54 (N=252) | 7 | 2.7(1.2-5.6) |  | 37 | 15.6(11.4-20.8) |  | 27 | 10.3(7.2-14.8) |  | 19 | 7.7(4.9-11.8) |  | 28 | 11.4(7.9-16.1) |  |
| 55-64 (N=152) | 4 | 2.5(0.9-6.7) |  | 23 | 15.7(10.6-22.8) |  | 14 | 8.5(5.1-14.1) |  | 4 | 2.6(0.9-7) |  | 8 | 5.6(2.8-10.9) |  |
| 65+ (N=165) | 5 | 2.5(1-6.1) |  | 9 | 5.5(2.9-10.4) |  | 13 | 6.7(3.9-11.4) |  | 4 | 3(1.1-7.7) |  | 18 | 11.4(7.3-17.2) |  |

*Weighted percentages to account for survey design

| **Socio-demographic**  **characteristics** | **Scoff Sick**  **(Item 1)** | | **Scoff Loss of Control**  **(Item 2)** | | **Scoff weight loss**  **(Item 3)** | | **Scoff fear of fatness**  **(Item 4)** | | **Scoff food thoughts**  **(Item 5)** | |
| --- | --- | --- | --- | --- | --- | --- | --- | --- | --- | --- |
|  | **Crude OR**  **(95% CI)** | **Adjusted OR§**  **(95% CI)** | **Crude OR**  **(95% CI)** | **Adjusted OR§**  **(95% CI)** | **Crude OR**  **(95% CI)** | **Adjusted OR§**  **(95% CI)** | **Crude OR**  **(95% CI)** | **Adjusted OR§**  **(95% CI)** | **Crude OR**  **(95% CI)** | **Adjusted OR§**  **(95% CI)** |
| **Gender** |  |  |  |  |  |  |  |  |  |  |
| Male (N=714) | 1.0 | 1.0 | 1.0 | 1.0 | 1.0 | 1.0 | 1.0 | 1.0 | 1.0 | 1.0 |
| Female (N=930) | 1.1(0.63-2.03) | 1.1(0.6-1.99) | 2.1(1.5-2.9)** | 1.9(1.4-2.8)** | 0.7(0.5-0.89)* | 0.6(0.43-0.79) | 2.4(1.53-3.67)** | 2.5(1.58-3.93)** | 1.4(0.99-2)* | 1.4(0.94-1.97) |
|  |  |  |  |  |  |  |  |  |  |  |
| **Ethnicity** |  |  |  |  |  |  |  |  |  |  |
| *White* (N=1,024) | 1.0 | 1.0 | 1.0 | 1.0 | 1.0 | 1.0 | 1.0 | 1.0 | 1.0 | 1.0 |
| *Black*  (N=361) | 0.9(0.42-2.29) | 0.9(0.42-2.3) | 1.5(1-2.08)* | 0.9(0.63-1.36) | 1.6(1.16-2.34)* | 1.2(0.83-1.81) | 1.4(0.87-2.21) | 1.1(0.65-1.81) | 1.2(0.76-1.83) | 0.9(0.61-1.56) |
| *Asian* (N=60) | 3.1(1.16-8.48)* | 3.7(1.36-9.85)* | 2.2(1.15-4.12)* | 2.2(1.14-4.22)* | 0.6(0.2-1.57) | 0.5(0.17-1.46) | 1.6(0.53-4.81) | 1.5(0.46-4.85) | 3(1.53-5.9)* | 3.1(1.54-6.28)* |
| *Other* (N=197) | 2.8(1.4-5.5)* | 2.7(1.36-5.39)* | 1.6(1.02-2.42)* | 1.3(0.83-2) | 0.9(0.51-1.48) | 0.7(0.38-1.15) | 2.7(1.59-4.67)** | 2.2(1.24-3.88)* | 2.4(1.56-3.84)** | 2.4(1.47-3.79)** |
|  |  |  |  |  |  |  |  |  |  |  |
| **Body Mass Index (BMI)** |  |  |  |  |  |  |  |  |  |  |
| *Underweight* (N=39) | 1.5(0.35-6.3) | 1.5(0.33-6.81) | 1(0.29-3.46) | 1.1(0.3-4.17) | 0.4(0.09-2.27) | 0.4(0.07-2.17) | 0.8(0.24-2.69) | 0.9(0.31-3.19) | 1.6(0.61-4.39) | 1.7(0.67-4.51) |
| *Normal weight* (N=708) | 1.0 | 1.0 | 1.0 | 1.0 | 1.00 | 1.0 | 1.0 | 1.0 | 1.0 | 1.0 |
| *Overweight* (N=506) | 0.5(0.25-1.12) | 0.5(0.28-1.24) | 1.5(1.03-2.27)* | 1.9(1.25-2.84)* | 0.9(0.65-1.37) | 1.1(0.71-1.56) | 0.8(0.51-1.31) | 0.9(0.59-1.6) | 1.2(0.8-1.84) | 1.4(0.89-2.08) |
| *Obese* (N=332) | 0.9(0.46-2.04) | 1.1(0.51-2.53) | 2.5(1.7-3.65)** | 3.2(2.15-4.87)** | 1.4(0.92-2) | 1.6(1.03-2.52)* | 0.64(0.34-1.19) | 0.7(0.36-1.44) | 1.8(1.16-2.81)* | 1.9(1.18-3.17)* |
|  |  |  |  |  |  |  |  |  |  |  |

**Table S3:** Crude and adjusted Odds ratios (ORs) and 95%CIs of the association between ethnicity and BMI and individual SCOFF answers***

§ Adjusted for age, gender, BMI, marital status, ethnicity, and education

* p ≤ 0.05

** p≤ 0.0001

*** multiply imputed models
